# *AIP* variant causing familial prolactinoma

**DOI:** 10.1007/s11102-020-01085-5

**Published:** 2020-10-03

**Authors:** David M. Carty, Rachael Harte, Russell S. Drummond, Rebecca Ward, Kesson Magid, David Collier, Martina Owens, Márta Korbonits

**Affiliations:** 1 Department of Diabetes, Endocrinology & Clinical Pharmacology, Glasgow Royal Infirmary, Glasgow, UK; 2 University of Exeter Medical School, Exeter, UK; 3 Centre for Endocrinology, William Harvey Research Institute, Barts and the London School of Medicine and Dentistry, Queen Mary University of London, London, UK; 4 Exeter Genomics Laboratory, Royal Devon and Exeter NHS Foundation Trust, Exeter, UK

**Keywords:** Prolactinoma, AIP mutation, Familial, Pituitary

## Abstract

Pathogenic variants in the aryl hydrocarbon receptor-interacting protein (*AIP*) gene are increasingly recognised as a cause of familial isolated pituitary adenoma. *AIP*-associated tumours are most commonly growth hormone (GH) producing. In our cohort of 175 *AIP* mutation positive patients representing 93 kindreds, 139 (79%) have GH excess, 19 have prolactinoma (17 familial and 2 sporadic cases) and out of the 17 clinically non-functioning tumours 4 were subsequently operated and found to be GH or GH & prolactin immunopositive adenoma. Here we report a family with an *AIP* variant, in which multiple family members are affected by prolactinoma, but none with GH excess. To our knowledge this is the first reported family with an *AIP* pathogenic variant to be affected solely by prolactinoma. These data suggest that prolactinoma families represent a small subset of *AIP* mutation positive kindreds, and similar to young-onset sporadic prolactinomas, *AIP* screening would be indicated.

## Introduction

Although most pituitary tumours arise sporadically, in around 5% of cases there is a familial presentation. Familial isolated pituitary adenoma (FIPA) is defined as pituitary tumours occurring in two or more family members, in the absence of other recognised genetic syndromes [1]. Pathogenic variants in the aryl hydrocarbon-interacting protein gene (*AIP*) have been increasingly recognised since their initial description in 2006, and are reported in up to 15% of FIPA families [2]. Pituitary tumours affect 17–23% of individuals with a pathogenic *AIP* variant [3, 4]. *AIP* associated tumours are most commonly growth hormone (GH) producing; presenting at a younger age with large tumours that are relatively resistant to conventional medical therapy, with more male patients recognised [2]. Around 10% of *AIP*-related pituitary tumours are solely prolactin producing [5, 6]; all reported prolactinoma cases have either had family members with GH excess or were simplex cases of *AIP* mutation positive sporadic prolactinomas. Here we report a family with homogenous familial prolactinoma segregating a pathogenic *AIP* nonsense variant.

## Case 1

**Fig. 1:**
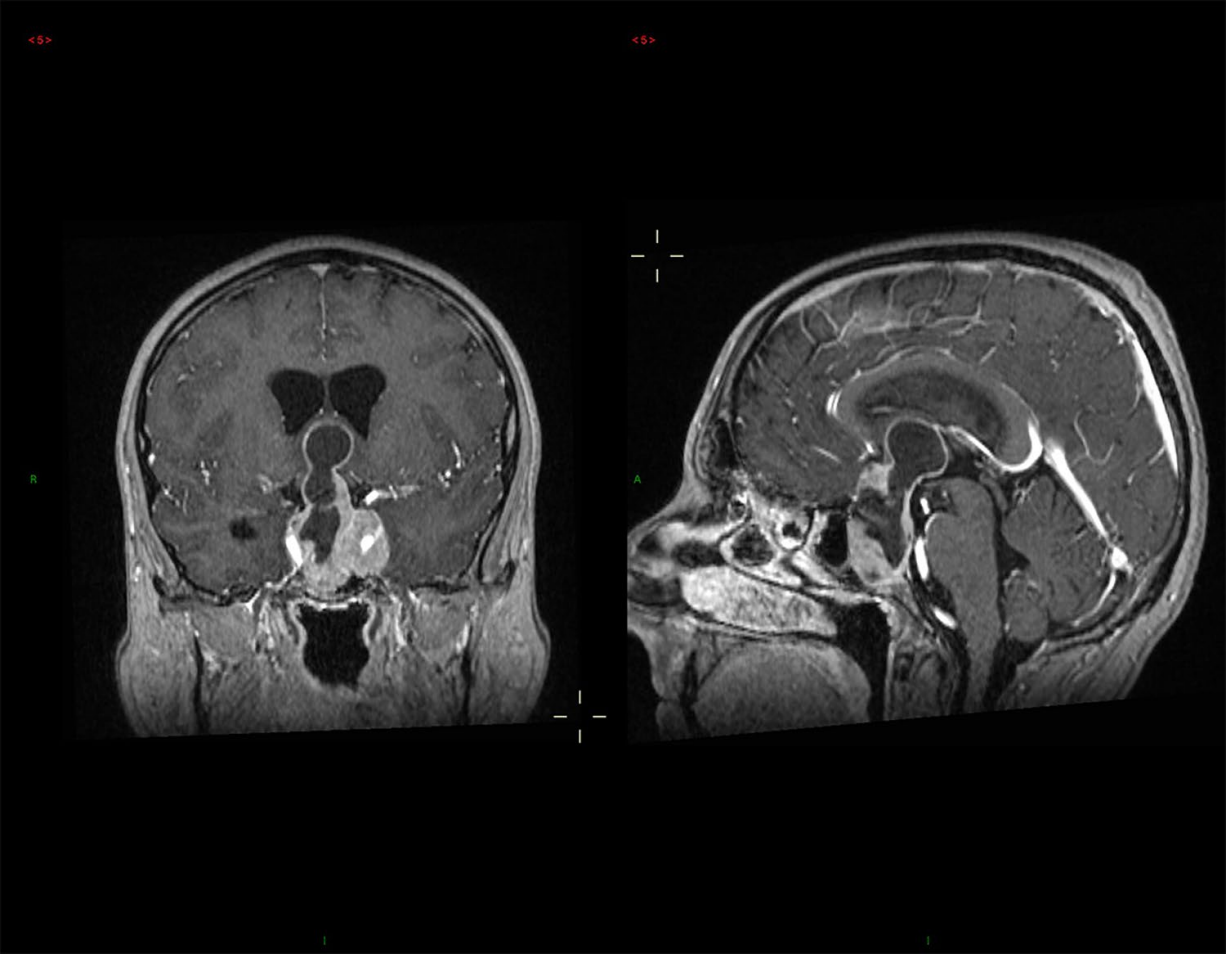
Coronal (left) and sagittal (right) MRI images pre treatment

**Fig. 2:**
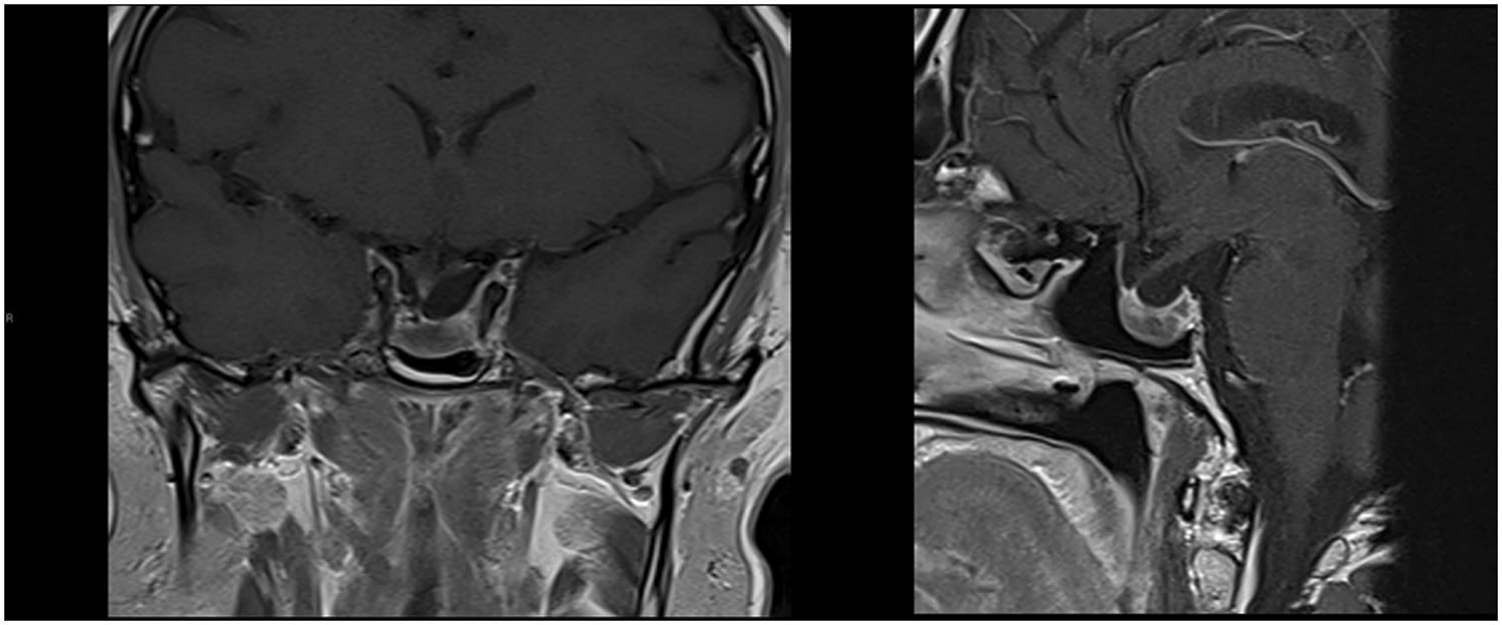
Coronal (left) and sagittal (right) MRI images following treatment

A 46-year-old man presented in 2012, having been referred from ophthalmology with a 6-month history of visual blurring; Goldmann perimetry demonstrated bitemporal hemianopia. In retrospect, he also gave an approximately 6-month history of erectile dysfunction and 1-month history of headache. Biochemical testing revealed a serum prolactin of 199,490 mU/l (male reference range < 400 mU/l) and panhypopituitarism with serum testosterone 0.9 mmol/l (ref > 10), morning cortisol 60 nmol/l (ref > 250), free T4 10 pmol/l (ref 10–21) and IGF-1 27 µg/l (age-adjusted reference range 50–315). MRI scanning (Fig. 1) showed a large partly cystic, partly solid mass measuring 61 × 38 × 24 mm; fluid levels within the cystic component were felt to be suggestive of haemorrhage and blood breakdown products. His growth and pubertal development had been normal, and his final adult height is 178 cm (mid-parental height 177.5). He is unmarried and has never tried for children. He was treated with dopamine agonist therapy and at 7 years of follow-up he remains on 2 mg cabergoline weekly along with hydrocortisone, thyroxine and testosterone therapy. He has been unable to tolerate higher doses of cabergoline because of nausea. Imaging has shown significant reduction in tumour volume from an estimated 28 ml at diagnosis to 1 ml in 2019 (Fig. 2); latest prolactin level is 5892 mU/l, and he has a minor ongoing visual field impairment.

A family history of pituitary disease was elicited in 2017, and targeted sequencing of the *AIP* gene identified a heterozygous nonsense variant, NM_003977.3:c.910C>T p.(Arg304Ter), in exon 6.

## Case 2

His sister, at the age of 36 years, was referred to a different endocrine clinic in the same city in 2006 with a 9-month history of secondary amenorrhoea and galactorrhoea, which persisted after stopping citalopram. Her height is 167 cm (mid-parental height 163.5). Serum prolactin was elevated at 4437 mU/l (female reference range < 630 mU/l). MRI scanning revealed 2 discrete pituitary lesions measuring 9 and 7.5 mm respectively. She was treated with cabergoline; menses returned and galactorrhoea ceased. Other pituitary function was normal, including normal IGF-1. Since then she has remained on cabergoline 1 mg weekly, latest prolactin is 929 mU/l at 13 years of follow-up, imaging in 2019 (CT because of claustrophobia) has shown a single 8.5 mm lesion. She was found to be heterozygous for the same *AIP* variant in 2017.

She had two children when she was 25 and 27 years old: her son (Case 3) has the *AIP* variant, while her daughter did not inherit the variant.

## Case 3

The 19-year-old son of Case 2 with the pathogenic *AIP* variant was identified on family screening in 2018. Growth and pubertal development had been normal and height at initial review was 180 cm (mid-parental height 176 cm). Prolactin was elevated at 2,131 mU/l in 2018 with morning testosterone of 7.7 nmol/l (reference > 10 nmol/l), IGF-1 was 210 µg/l (age adjusted reference range 105–410) and MRI showed a 7 mm left-sided pituitary adenoma. He was treated with cabergoline initially 0.5 mg then 0.75 mg weekly. Imaging shows the lesion has not changed in size at 18 months of follow-up; prolactin is 807 mU/l, his morning testosterone normalised to 15.8 nmol/l and cabergoline is being up-titrated.

## Case 4

A 36-year-old second sister of Case 1 was referred in 2007 with a 2 year history of secondary amenorrhoea. She had a significant history of anxiety and learning difficulties. Her height is 158 cm (mid-parental height 163.5 cm). Serum prolactin was 3936 mU/l which was felt, at least in part, to be related to anti-psychotic and antidepressant medications. She was unable to undergo MRI imaging due to claustrophobia, but in 2008 underwent CT pituitary which demonstrated an 8 mm left-sided microadenoma. Remaining pituitary function was normal. Prolactin levels have remained elevated at 1685 mu/l at 12 years of follow-up despite 2 mg weekly cabergoline therapy; imaging in 2019 has shown stable appearances and she has been unable to tolerate higher doses of cabergoline due to mood disturbance. She is heterozygous for the same *AIP* variant.

**Fig. 3:**
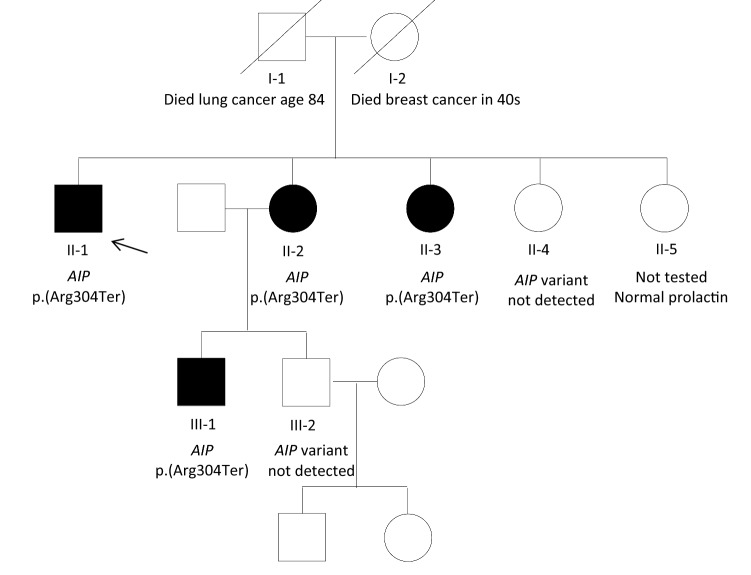
Pedigree. Filled symbols represent family members with a prolactinoma. Proband (case 1) is indicated by the arrow. Square symbols: male, circles: female

There are two remaining female siblings (Fig. 3); one did not have the *AIP* variant, the other has not given her consent for genetic testing but has a serum prolactin within the reference range and she is of normal height.

## Discussion

A number of genetic syndromes are implicated in hereditary pituitary tumours, including Multiple Endocrine Neoplasia Types 1 and 4, Carney Complex, X-linked acrogigantism and McCune-Albright syndrome. Pathogenic variants in the *AIP* gene are now increasingly recognised in families with isolated pituitary tumour syndrome, typically causing gigantism, with children as young as 4 years being reported to be affected. Pathogenic *AIP* variants have also been identified as founder variants, such as the one in Northern Ireland, arising over 100 generations ago and being responsible for numerous current and several historical Irish giant patients [3, 7]. Here we report a Scottish family, who are not known to be of Irish descent, with a history of prolactinoma. There is no family history of gigantism or acromegaly, and all affected patients had an IGF-1 within the age-adjusted reference range. We have identified 2 additional families with the same *AIP* variant born in the same city.

Much of the literature around *AIP* variants is focused on GH excess. Our large series of cases with *AIP* variants identified 10% of patients with prolactinomas, while tumours with negative GH and prolactin staining are exceedingly rare [6]. It is currently unclear why mutations in the widely expressed *AIP* gene leads to tumorigenesis in only somatotrophs and lactotrophs rather than other members of the PIT1 linage, other pituitary cell types or other organs. It is also uncertain why in this family, uniquely, none of the affected members developed GH excess, although, since none of the four affected family members underwent surgery, immunohistochemical staining for GH has not been undertaken. The patient with the macroadenoma (Case 1) had no history of recent sudden-onset headache at diagnosis, but MRI features were compatible with haemorrhage which could be related to pituitary apoplexy, often described in *AIP* mutation positive cases [5, 6, 8–11]. Ongoing research using various animal models may help to explain these clinical observations in the future.

GH-secreting *AIP* mutation positive tumours are typically resistant to first generation somatostatin analogues, but the responsiveness of *AIP* mutation positive prolactinomas to dopamine agonists remains unclear. In our patients, although prolactin levels reduced in all 4 subjects, none showed normalisation of prolactin levels at the doses patients were able to tolerate. Based on the lack of normalisation with < 2 mg cabergoline/week criteria [12], 2 of our 4 *AIP* mutation positive patients are resistant, while the other 2 cannot be fully assessed as they have not reached this cabergoline dose. Regarding tumour shrinkage, 3 out of the 4 patients showed reduction (but not disappearance) of tumour size, while there was no change in tumour size in one patient. These data indeed suggest a level of dopamine agonist resistance both for prolactin level and for tumour shrinkage.

In respect to other reported *AIP* kindreds, it is relevant that in this family there were 2 males and 2 females affected, with 3 of the 4 patients having a microadenoma (although one was diagnosed prospectively). In our overall cohort of 175 *AIP* mutation positive patients representing 93 kindreds, 139 (79%) have GH excess, 19 have prolactinoma (17 familial and 2 sporadic cases) and out of the 17 clinically non-functioning tumours 4 were subsequently operated and found to be GH or GH & prolactin immunopositive adenoma. Out of 19 *AIP* mutation positive prolactinoma patients with a mean age of diagnosis of 29 years in our full *AIP* mutation positive cohort (including cases from this family) [6], there were 10 female patients, 12 with macroadenomas and 10 had surgery. An earlier study reported that of 13 *AIP* mutation positive prolactinoma patients with a mean age of diagnosis of 22 years, 10 were males, 12 had macroadenoma and 6 had surgery [2]. Among the 3 prolactinoma patients in a paediatric prolactinoma cohort with unequivocally pathogenic *AIP* variants, 2 were males and all 3 had invasive macroadenomas with a mean age of diagnosis at 16 years [13].

To our knowledge this is the first family with a pathogenic *AIP* variant to be described who are affected solely by prolactinoma. These data suggest that pure prolactinoma families represent a small subset of *AIP* mutation positive kindreds. Genetic screening for *AIP* mutations is advised both in familial or sporadic young-onset prolactinoma and GH excess cases, as early diagnosis in family members leads to better clinical outcomes [6].
